# Audiovisual communication of object-names improves the spatial accuracy of recalled object-locations in topographic maps

**DOI:** 10.1371/journal.pone.0186065

**Published:** 2017-10-23

**Authors:** Nils Lammert-Siepmann, Anne-Kathrin Bestgen, Dennis Edler, Lars Kuchinke, Frank Dickmann

**Affiliations:** 1 Geomatics/Cartography Group, Geography Department, Ruhr-University Bochum, Germany; 2 Methodology and Evaluation, International Psychoanalytical University Berlin, Germany; University of Sussex, UNITED KINGDOM

## Abstract

Knowing the correct location of a specific object learned from a (topographic) map is fundamental for orientation and navigation tasks. Spatial reference systems, such as coordinates or cardinal directions, are helpful tools for any geometric localization of positions that aims to be as exact as possible. Considering modern visualization techniques of multimedia cartography, map elements transferred through the auditory channel can be added easily. Audiovisual approaches have been discussed in the cartographic community for many years. However, the effectiveness of audiovisual map elements for map use has hardly been explored so far. Within an interdisciplinary (cartography-cognitive psychology) research project, it is examined whether map users remember object-locations better if they do not just read the corresponding place names, but also listen to them as voice recordings. This approach is based on the idea that learning object-identities influences learning object-locations, which is crucial for map-reading tasks. The results of an empirical study show that the additional auditory communication of object names not only improves memory for the names (object-identities), but also for the spatial accuracy of their corresponding object-locations. The audiovisual communication of semantic attribute information of a spatial object seems to improve the binding of object-identity and object-location, which enhances the spatial accuracy of object-location memory.

## Introduction

Cognitive representations of geographic space (*cognitive maps*) are processed and coded in spatial memory. Given that cognitive maps are internal representations of geographic space, they are influenced through interactions with the external world [1]. Spatial details contained in a cognitive map are predominantly learned in the ‘real’ environment (*direct experience* or *primary learning*). Direct experience is a multisensory way of learning spatial information, including visual but also auditory, haptic or olfactory inputs [2–4]. As alternative ways, spatial information can be taken up through verbal descriptions [5–7] or cartographic media representing the environment, i.e. maps or map-like visualizations. Indirect experience through maps [8–10] occurs in many situations of everyday life when spatial choices and decisions are to be made, e.g. route planning, hotel search, navigation, geography teaching, outdoor activities etc.

An essential aspect of cartographic media is the amount of different information a map user is confronted with. A topographic map, for instance, provides information about spatial objects, such as their geometric and semantic properties, about spatial arrangement (patterns) and also about their position within a spatial reference system. All these aspects contribute to the formation of cognitive representations of space [11]. In the process of building a cognitive map with access to all this information simultaneously, the map user is able to filter and connect certain information to construct an effective cognitive map. The ability to encode and later on to recall sufficient spatial information from the cognitive map is the fundamental basis for spatial orientation and navigation. The formation of a cognitive map depends on the design of map graphics and on the hierarchical structure of spatial memory organization [12, 13].

The processing of object properties, object location and the configuration of objects involves different memory processes. For using topographic maps, it is required that the exact location of an object is processed in combination with its other semantic properties. Storing both types of information with reference to each other requires an object-location binding. Binding can be more difficult in cases where property information is very complex [14, 15]. The complexity of semantic information may thus influence the accuracy of object-location memory.

## Distortions in object-location memory

Maps are visual media that are commonly understood to communicate spatial information graphically [16–18]. Thus, the configuration of graphically represented spatial information plays an important role for map reading and spatial decisions made afterwards. Learning spatial information through graphics, however, is influenced by distortion tendencies decreasing orientation and navigation performance. Studies demonstrate that spatial information learned from cartographic media is substantially changed on its way from the original source to subsequent memory recall [12, 19, 13]. Obviously, the topological relation of spatial object positions being mapped and their supposed representation within the user’s mind is not identical [1]. Original spatial information can be strongly distorted, which leads to inaccurate estimates of spatial configurations, such as distances or angles [20].

Theoretical principles of cartography are intended to enhance the information transfer in cartographic communication. This means that, for instance, distortion tendencies in spatial memory should be reduced as much as possible. To achieve this, one possibility is offered by the characteristics of the distortions themselves, as distortions mainly follow systematic and predictable structures [11, 21–24]. If such predictable structures of spatial distortions are being considered right at the beginning of making a map, characteristic errors in location memory can be avoided.

Studies show that the map user’s object-location memory performance can be improved by implementing specific graphic features [25–27]. Such graphic changes refer to additional grids or linear symbols. Projecting a road network closer to the viewer in a 3D map (based on autostereoscopic displays and depth effects) increases both the salience of the linear symbols representing the roads and, even more importantly in terms of map use, the attention that viewers pay to them and their structure. In addition to the use of graphic symbols in cartography, technical developments allow the addition of information in other modalities, such as in the auditory dimension. Since the 1990s, it has been suggested many times it would be interesting to explore cartographic use of sound and to investigate its impact on map use (e.g. [28–38]). Multimedia cartographic techniques and software can address and implement multiple sensory channels, especially the visual and auditory channels. The use of both channels influences cartographic information transfer and map use, which may influence the formation of cognitive maps [39, 40]. In order to investigate the impact of audiovisual communication in maps on spatial memory, it is, first of all, important to have a closer look at the conditions of this multimodal approach.

## Auditory modality in spatial cognition

Advantages of multisensory integration have been reported by several different cognitive psychology studies. The integration of information from different sensory modalities is known to increase detection thresholds [41], attention towards stimuli [42] and the recognition of related information [43]. The multiple resource theory [44] points to the advantage of facilitated processing of information from different modalities instead of processing information from a single modality. The information from another modality provides additional contextual information, which leads to a combined retrieval cue [45], while matching contextual information during encoding improves the retrieval performance [46]. The integration of audiovisual information was the focus of a number of studies [47–50]. The memory performance for auditory stimuli in contrast to visual stimuli is inferior [47]. However, there is evidence of very early integration of audio and visual information, which leads to an enhanced perception of this multimodal information as one combined single percept [48].

The use of the auditory modality might improve spatial information transfer, such as in navigation systems where auditory guidance is provided in addition to the visual map. Auditory elements (often computer-generated verbal instructions) are common design features used as add-on verbal instructions in car vehicle navigation systems [49, 50]. It has been shown that this additional auditory signal accelerates spatial orientation performance on a display; already Dinh et al. [51] provided empirical evidence that additional sensory input does not only increase the sense of, but also the memory for objects in a virtual environment. Furthermore, when landmarks are provided with verbal instructions within auditory route guidance systems, significantly fewer navigational errors were reported in subsequent memory tests [49].

An important aspect however was shown in studies on the integration of visual and auditory features (dual coding effect) [39, 40, 52–54]. Using more than one sensory modality does not necessarily lead to a simple increase in transferred information. The information transfer is also dependent on the peculiarities of the different modalities. Thus, their different impacts on cognition must be considered. For example, it was shown that transferring information audiovisually (verbal redundancy: spoken and written text) improves memory performance, but only if the information to-be-learned is short—up to one sentence, according to the capacity of the phonological loop (c.f. [55]). In terms of more complex information, audiovisual redundancy causes disadvantages over a written text approach [56–58]. This matches a general assumption of “Cognitive Load Theory” (CLT, [59, 60]), since the cognitive processing of more complex information requires a higher working memory capacity. Accordingly, a higher amount of cognitive capacity (germane load) needs to be invested into creating a permanent storage of knowledge.

In cartography, the modality effect may be used for the audiovisual communication of route directions in navigation systems. Such instructions have a manageable length, which would probably not lead to cognitive overload. So far, effects on spatial memory performance based on map learning have been mainly investigated through the visual sensory modality [61, 62, 51]. An increasing number of publications on audiovisual cartography within the last decades (overviews in [30, 63]) shows that the addition of auditory map features is generally regarded as a cartographic design approach that is appropriate for improving the map-based communication of spatial information.

Auditory cues that are likely to enhance spatial memory performance are rarely implemented in map-based studies of spatial cognition [64]. Research should focus more on the effective application of additional auditory information and an analysis of how auditory and visual spatial information interact in spatial memory.

## Cross-modal object-information

Using animation software and standard techniques of multimedia cartography, auditory elements, such as abstract sounds, recordings of the real sonic environment (audiorealistic “soundscape”, see [65]), vocal narration and music, could easily be integrated into digital maps [66, 63, 33, 67, 68]. Voice narration is the most obvious sound to represent distinct semantic attributes of map objects [16, 31]. So far, its impact on spatial memory has rarely been in the focus of research.

In order to implement auditory map elements effectively, it needs to be explored whether their use would additionally support spatial memory. If so, they could be used to reduce distortion errors in cognitive maps.

According to cognitive psychology, spatial information consists of three main components: object information (identity), positional information (location) as well as a third component (object-to-location binding) which connects identity and location [14, 15]. Recent studies support the assumption that information about an object-location and object identity is held separately. For instance, location information is not connected with the recalled object [69], and the recall of a location seems to be superior to the recall of object-identity [70]. An object-location can be recalled without the object identity information. Nevertheless, the binding of location and identity is fundamental for an effective cognitive map, as it helps to process and remember the position of environmental objects as well as their meaning. For both object properties to be remembered, they have to be bound together, and recalling bound information is more difficult than remembering the object identity or object location alone [71, 15].

There is an ongoing debate about storage of these object properties in memory. It is discussed that a specific episodic buffer is specialized in holding object-locations bindings [72, 73]. Furthermore, object-location-binding is modulated by time and by the number of objects [71] and binding failures decrease the performance of object-location memory [15].

According to the theory of multimedia learning [58, 57], the cross-modal presentation of spatial information has a mainly beneficial impact on spatial memory. Therefore, we expect a positive effect of audiovisual communication of object-names on object-location-binding. Object-location binding should benefit from an enhanced object-identity processing by the audiovisual communication of object-names. Following this direction, Lammert-Siepmann et al. [31] report that simultaneous auditory and visual communication of place names in maps improves memory performance of object identity. It still remains an open question whether the auditory map elements would generally support object-location binding and, thus, object-location memory performance. Would the use of auditory map elements referring to semantic attributes of an object, such as voice recordings of place names, help to recall the geometric position of a learned object-location more accurately?

## Study on audiovisual communication in maps on object-location memory

To address this question in an experimental design, a recall memory paradigm was used (for other recall studies in cartography, see [74, 27, 75, 36]). In this study, subjects were asked to learn (encode) object-locations and object-names and to recall them after a filler task. The recall was measured by the percentage of correctly recalled object-names and locations (hit rate) and the average deviation from the original object-location (spatial accuracy).

## Experimental Procedures

### Methods

#### Participants

Thirty-two participants (11 female, 21 male) aged between 18 and 31 (M = 24.6; SD = 3.8) took part in the study for pay. All participants were unaware of the study’s purpose and reported having normal or corrected-to-normal hearing and vision. All participants were students at the Ruhr-University Bochum (RUB). The participants were unfamiliar with the topographies represented in the study materials. The study was conducted in accordance with the Declaration of Helsinki and was approved by the local ethics committee of the Faculty of Psychology, Ruhr-University Bochum (Germany). All participants gave their written informed consent before being included in the study.

#### Stimuli

Referring to cartographic materials used in previous studies of cartography and spatial cognition (cf. [76, 27]), six different multi-coloured digital topographic maps were created as study materials. They include all layers of the present digital topographic map of North Rhine-Westphalia, Germany (DTK10-NRW). Any regular verbal elements, such as written street names, were removed from the maps in order to avoid associations and other memory effects. The scale of each map is 1/10,000. This map scale is officially recommended for users dealing with, for instance, route planning, travel management, city maps and tourism, i.e. user groups concerning wayfinding and navigation issues. The map size is 1065 px * 710 px (30 cm * 20 cm).

**Fig 1:**
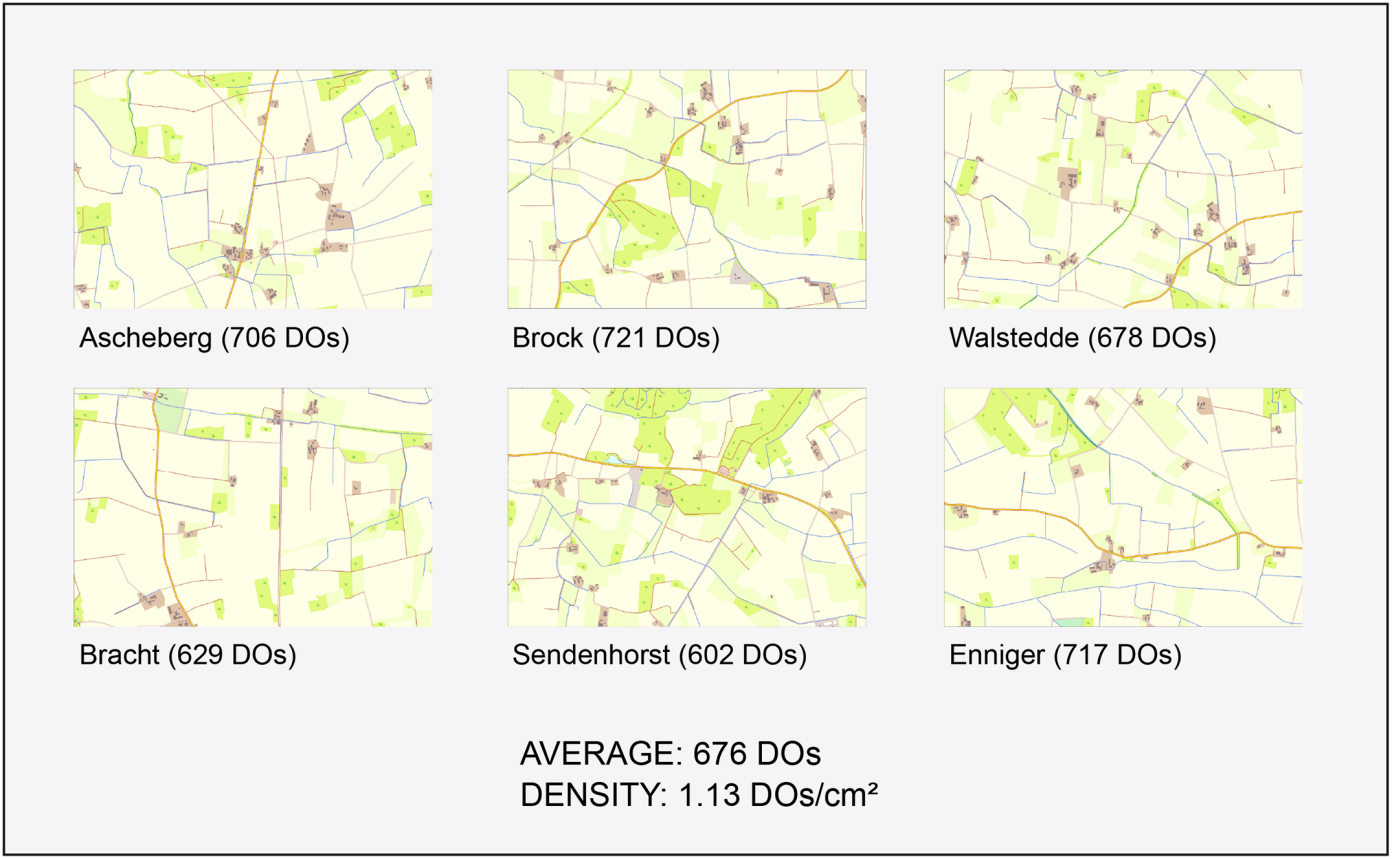
Overview of maps used as experimental stimuli. The six urban maps used as test materials. Each map (scale used in the experiment: 1:10,000) is derived from ATKIS^®^-Basis-DLM (data source: Geobasis NRW 2016). The maps represent rural topographies in North Rhine-Westphalia, Germany (see place names below the maps). The distinct objects (DOs) of each map are determined as a quantifying measure of map complexity. The average number of DOs and the object density (average DOs/600 cm^2^) are additionally determined.

The six maps are similar in terms of their topographic situation and average object density. They show a rural and flat topography. The average object density is measured using an object-oriented image (map) segmentation approach reported in Edler [77]. The average number of distinct map objects is 676, which leads to an average object density of about 1.13 distinct objects / cm^2^ (Fig 1).

The maps are additionally augmented with seven point symbols representing the locations of fictional places. The seven points are randomly selected from a set of 50 location options previously defined for each map. The Euclidian distance between the points is higher than 2 cm. All symbols are identical in size (d = 0.5 cm) and colour (R: 225, G: 0, B: 200).

Each of the seven randomly selected points (spatial information) is combined with a geographic name (semantic / attribute information) that is also randomly selected, from a pool of 42 possible options. The geographic names are fictional German place names consisting of eight characters, such as Landkoog, Sandkiez, Sonnfeld and Wallsund.

In three maps, the place name was communicated visually through written information (Arial, 11 pt., black). In the other half, the names were presented in an audiovisual way, i.e. written (Arial, 11 pt., black) and their spoken equivalents. In accordance with the voice principle of multimedia learning, the place names were spoken by a human voice and not computer-generated [78, 79]. As changes of the speaker can influence memory performance [80–82], all place names were recorded by the same male speaker. The assignment of the six maps to the **visual** and **audiovisual** conditions was randomized.

The sound files were recorded with high-quality devices. According to the capacity of the phonological loop [55], each recording has a maximum length of two seconds. To stay within the frame of two seconds, all place names were composed of eight characters and a maximum of three syllables. The recorded files were post-processed with a noisegate in order to eliminate any possible background noise. Based on an equalizer, the timbre is standardized. Finally, a compressor was used to normalize the audio files, which results in the same loudness across all the files.

The maps as well as the recorded sound files were embedded into a script tool based on *ActionScript* (v. 3.0), implemented in *Adobe*^®^ *Flash*^®^ *CS5*. This script was used to run the trials and to acquire all test data needed. The maps were displayed on a TFT-LCD 24” screen that was calibrated in order to represent the official colour scheme of the selected topographic maps.

#### Procedure

The study comprised a within-subjects design including the two conditions **visual** and **audiovisual**. Each of the thirty-six participants took part in six study-test trials in random order. In each trial, they were shown one of the six study maps for 60 seconds. The task in the first phase of the trial (encoding phase) was to learn both the locations and the names of the seven points.

**Fig 2:**
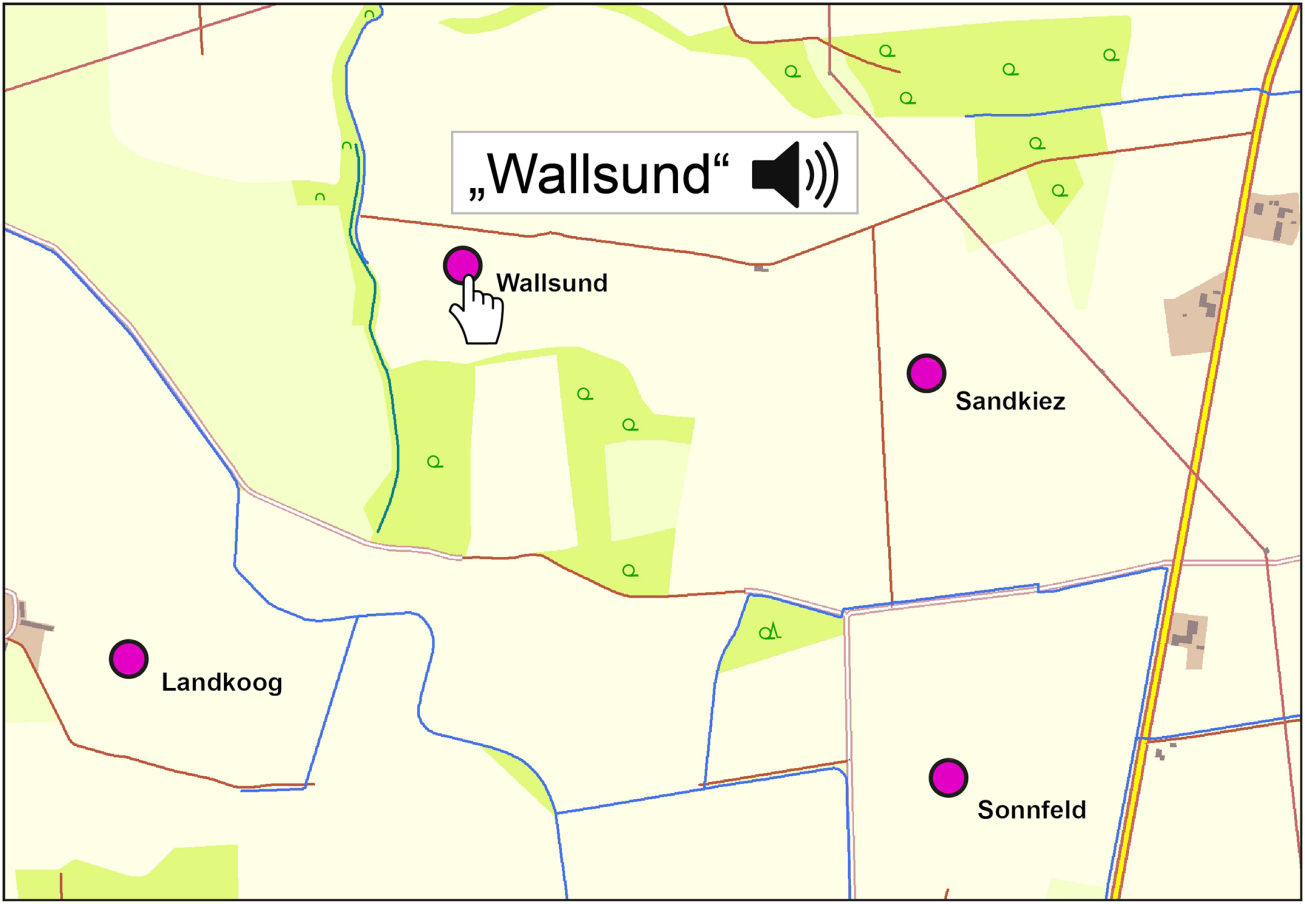
Activating the voice recordings of the place names in the audiovisual condition. The figure shows how the auditory map elements can be activated in the encoding phase of the test trials. The cursor is used to navigate to the circular symbols representing the object-locations. After clicking on the circles, the recordings of the place names are played. Repetitions are allowed.

In the **audiovisual** condition, the participants were instructed to play the recorded place names by clicking on the point symbols (Fig 2). The order was individually defined by the participants (free recall). Repetitions were possible. To receive the sound information, participants were given a pair of high-quality circumaural headphones (Sennheiser HD-201). To guarantee a proper and comfortable volume level throughout the study, the participants were asked to adjust the level before the beginning of the test. This individual adjustment was based on another set of three place names recorded and post-processed in the same way.

The encoding phase was immediately followed by a filler task (45 seconds of a task-independent search task). Finally, the map was shown again for 60 seconds without the seven points. The participants were requested to recall the seven object locations and names. To locate a point within the recall task, the participants were instructed to use the mouse cursor (recall phase). Having defined its location, a text input field automatically occured near the object. Both location and name had to be confirmed using a keyboard command. Corrections were possible within the given time limit. Before beginning with the six study-test trials, the original location participants were given a practice trial to become familiar with the software, the tasks, and the general test procedure. The participants were encouraged to complete the tasks as quickly and accurately as possible.

#### Statistics

The mean spatial memory performance was based on 1) the recalled written place names, and 2) the Euclidean distances between the x and y coordinates of the recalled objects and the corresponding original location coordinates.

The written text entries were rated and recoded as a binary quantity– 1 (correct) and 0 (incorrect). An entry was correct if the recalled name was identical with the original name. Only minor differences were also accepted as correct, such as obvious spelling mistakes, lower cases (first character) and examples where the written entry mirrored the correct pronunciation of the name (see “Sielstein” instead of “Silstein”–in both cases pronounced with a long German i-sound). The quantitative translation of the recalled place names further allows the calculation of a measure representing the mean percentage of correctly recalled object-names: *hit rate (names)*.

The Euclidean distance refers to the spatial deviation of the recalled object-location (centre) from its original position. The distance was measured in pixels (px). In accordance with previous research [83–85], the location of a recalled object-location was considered correct if it deviated no more than 35.5 px (0–1 cm) from the original location. The Euclidian metric in pixels was the quantitative base to statistically analyse the chosen measure of object-location memory, i.e. *spatial accuracy*: it represents the mean distance errors of correctly recalled object-locations. To focus on the analysis of object-location information combined with object identity information, the two location-based measures were also applied to data entries of correctly as well as incorrectly recalled object-names–*spatial accuracy (correct names)* and *spatial accuracy (incorrect names)*.

Paired-samples t-tests were applied to compare the means between the audiovisual and visual condition to analyse the percentage of correctly recalled names (hit rate (names)). The second analysis was applied to examine the spatial accuracy of the recalled object locations in combination with the identity information. A 2*2 within-subject ANOVA comprising the within-subjects factors modality (audiovisual vs. visual) and correct naming (correct vs. incorrect names; in the following referred to as “naming”) *was* computed. Because individual spatial accuracy values do not follow a gaussian distribution, a median aggregation for each subject was calculated to avoid distortions or bias by outliers.

The significance threshold was set at p = .05. Participants were excluded in the following cases:

1. The participant did not recall a single combination of object-location and object-identity (name) correctly across all six maps.
2. The participant did not recall one object-location each in at least three of the six maps.
3. The participant failed to recall at least 20% of correct object-locations in one of the two conditions (audiovisual or visual).

These criteria were put into place in order to ensure that participants were motivated enough to complete the test in the best possible way. Due to these constraints, the data of 32 (of 36) participants were considered in the statistical analyses.

### Results

#### Hit rate (names)

**Fig 3:**
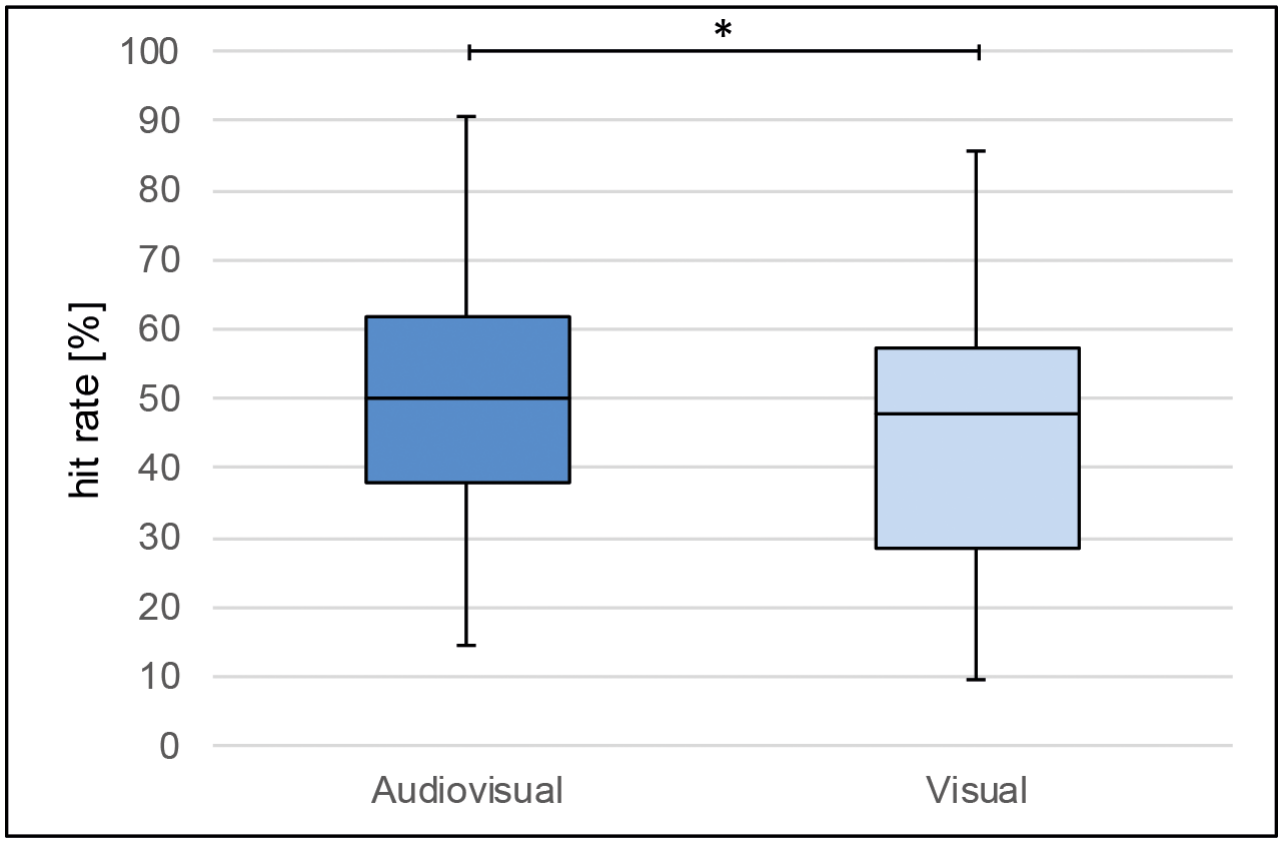
Differences of hit rate (names)–visual vs. audiovisual. **Hit rate** refers to the mean percentages of correctly recalled object-names (in %). * = p < .05.

The paired t-test on the mean percentages of correctly recalled object-names shows a significant difference between **audiovisual** (M = 50.15, SD = 16.67) and **visual** (M = 45.54, SD = 19.50; t(31) = 2.163, p = .038; ηp^2^ = .071) (Fig 3).

#### Spatial accuracy

**Fig 4:**
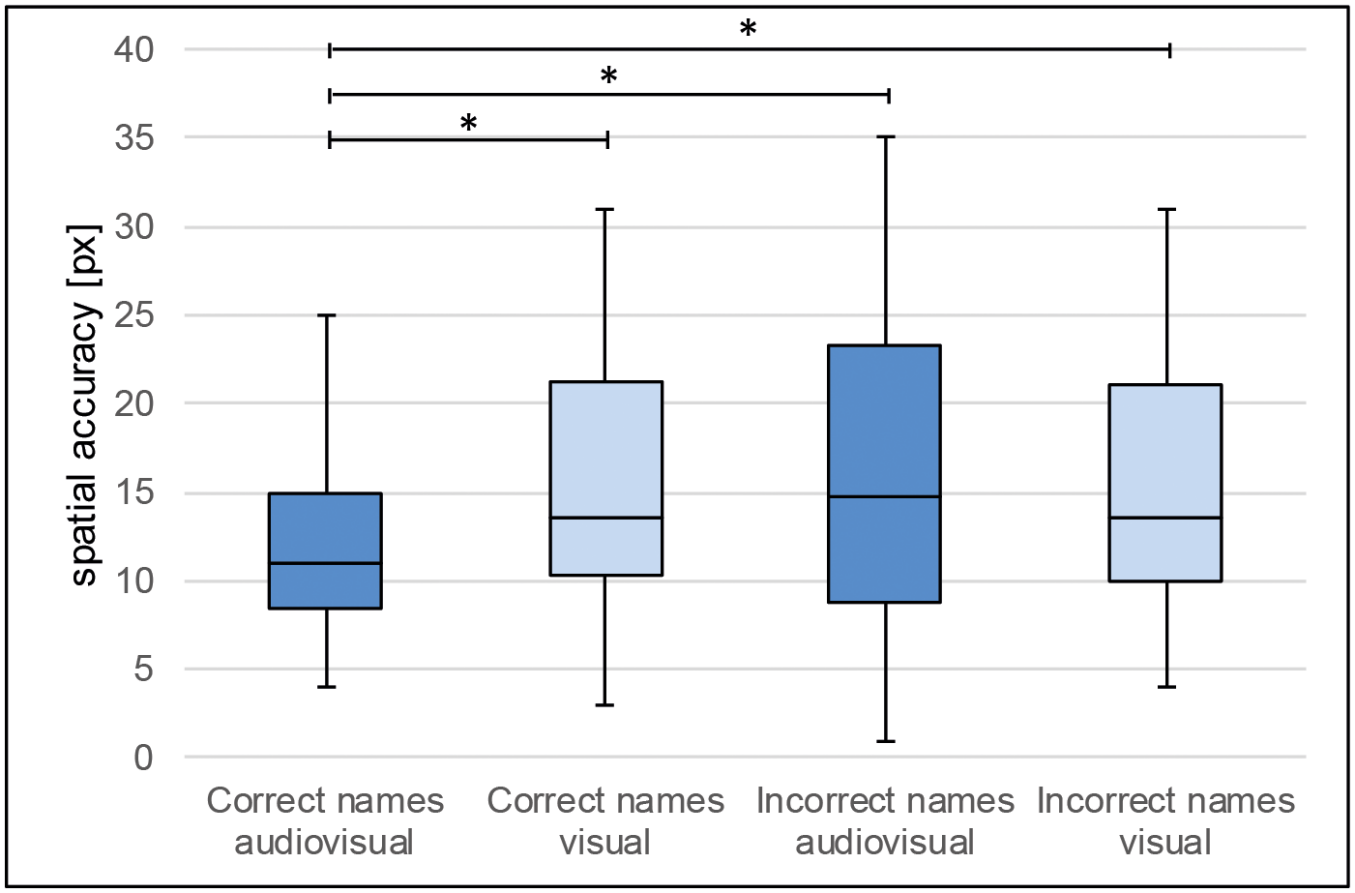
Spatial accuracy for audiovisual and visual conditions with correct and incorrect naming. Spatial accuracy refers to the mean distance errors of correctly recalled object-locations (in px). The recall of an object-location was regarded as correct if the recalled location was within a linear distance of 0–35.5 px (0–1 cm) from the location of the object to be learned.

Concerning the spatial accuracy performance, the repeated measures ANOVA reveals neither significant main effects of **modality** (F(1,31) = 1.330, p = .258, ηp^2^ = .041) nor of **naming** (F(1,31) = 1.876, p = .181, ηp^2^ = .057). But a significant modality * naming effect is visible (F(1,31) = 4.573, p = .040, ηp^2^ = .129). Multiple Bonferroni-corrected pairwise comparisons show that the interaction effect is based on a lower value (better performance) of spatial accuracy (i.e. an increase in spatial accuracy) in the **audiovisual** condition of correct names (M = 11.95), compared to the spatial accuracy in the **audiovisual** condition of incorrect names (M = 16.31; t (31) = -2.248, p = .032), the **visual** condition of correct names (M = 15.875; t (31) = -2.702, p = .011) and the **visual** condition of incorrect names (M = 15.30; t (31) = 2.05, p = .049; Fig 4). All other comparisons do not significantly differ from each other (all p’s > .05). It should be noted that all significant effects that were observed would still be present if the aforementioned 4 participants that were excluded were being considered as well.

### Discussion

The results of the present study point to the high potential of audiovisual approaches to communicate spatial information in maps. Firstly, the analysis of the hit rate for place names reveals a higher number of correctly recalled objects names after the names are communicated audiovisually (Fig 3). Object names presented as linked audiovisual information during encoding are remembered better than unimodal written place names (visual information only). Thus, as assumed before, the processing of object properties in two different sensory channels enhances the recognition of object-identity. This is in line with the results of previous studies [31, 61].

Even more interesting are the memory results of the spatial component, i.e. the object-location of the correctly recalled objects. The results point towards an effect of audiovisual integration during a spatial memory task. Audiovisual integration seems to facilitate object-location binding which was previously discussed to be the most difficult spatial memory process (e.g. [14]). An effect of modality becomes visible in terms of a significant modality*naming interaction; the main effect of modality on spatial accuracy itself is not significant. It is shown that a modality-based communication gears towards object-identity and not towards the object-location in a topographic map. Participants recall the location of map objects more accurately if they learn the objects’ identity information from an audiovisual presentation (written place names plus voice recordings of the names), and not solely in a unimodal written text form (Fig 4). Of particular note is that the objects referring to this spatial accuracy measure are correctly remembered by the participants, i.e. the full object information is available including both location and object-identity.

Another peculiarity is that there is no effect of modality on the spatial accuracy of incorrectly recalled object names. For incorrectly recalled object names, the difference between the spatial accuracy in the visual and the audiovisual condition is not significant. Additional auditory information does not generally enhance spatial accuracy. Thus, location information does not benefit from association with auditory information per se. Enhanced spatial accuracy can only be observed if correct identity information is available as well (as indicated by the significant interaction effect). We interpret this as evidence indicating the availability of two different types of information that are likely based on two separate underlying memory processes.

This effect provides further insight into the processing of object-location binding and corresponds to the findings reported by Pertzov et al. [71] who also identified an increased fragility of memory results when object-location and object-identity are linked in recall tasks. A likely explanation is that both types of object properties, correct identity and location, when stored separately from each other, need to be processed later as one representation that comprises location, identity and binding information for a successful recall of the object in space [15, 86, 87]. In the case of binding-failures, objects are not learned as a whole entity. Missing object-location binding seems to restrict the access to identity information in the present study. A separate recall of the object-location is still possible. It can be speculated that, without the unambiguous allocation to the object-identity, this information is useless for the most part (e.g. in the context of map navigation). Therefore, the activation of specific memory processes for object-location-binding is crucial, as it has been shown to be elicited by audiovisual information in the present study.

**Fig 5:**
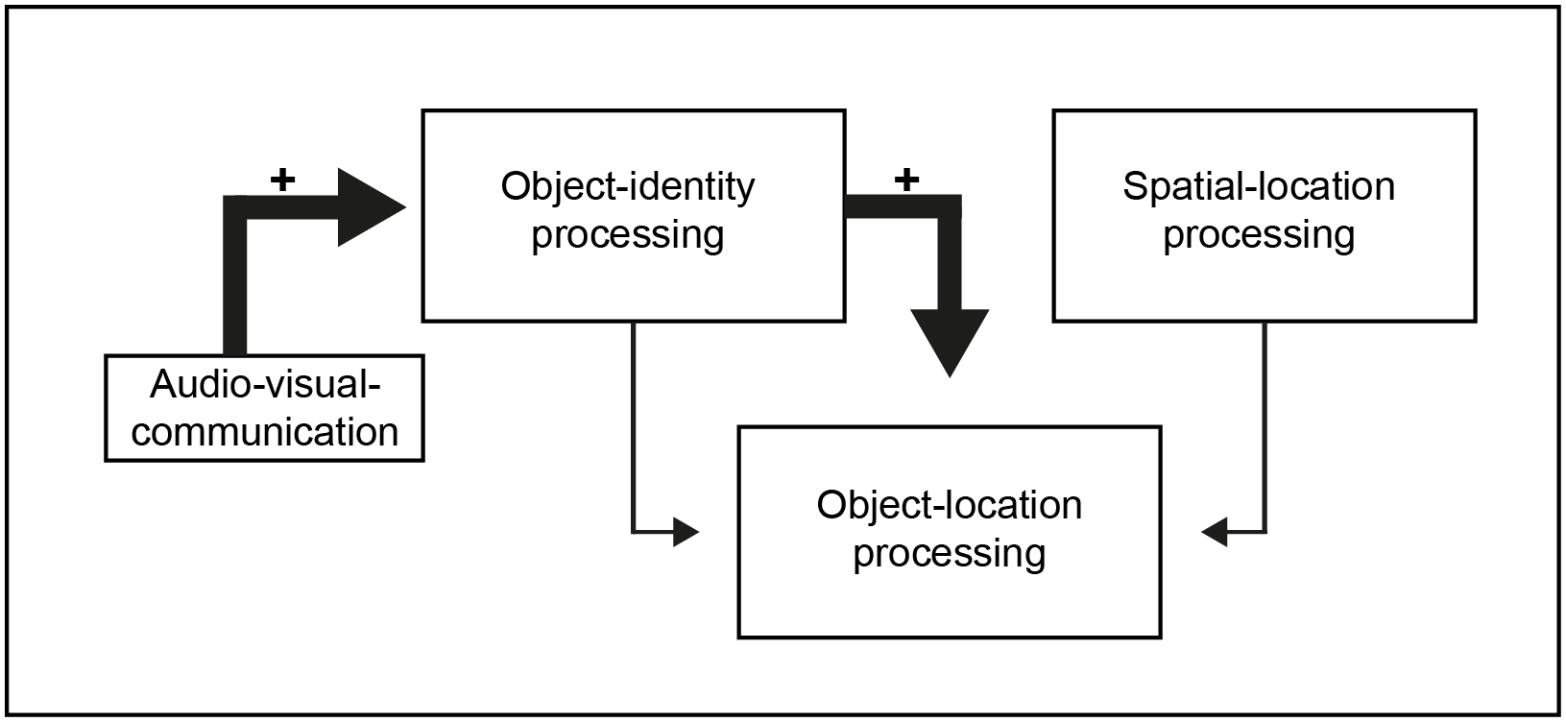
Schematic sketch of how object-location memory may be enhanced by audiovisual communication.

Previous results suggest that object-location-binding is modulated by time and number of items [71]. The present results expand these findings and reveal a modulation of object-location information by the modality the objects are presented with. Hence, using auditory (geographic) names in addition to the written names in topographic maps strengthens the object-identity processing, which supports object location memory via object-location binding (Fig 5).

### Summary and outlook

This study shows that audiovisual communication in multimodal maps can help to improve object-location memory performance. Auditory information, which can be used to define object identity, does not have an effect on spatial accuracy per se. This implies that object location is not affected by a feature that operates on object identity; object location memory benefits only when correct identity information is available. Thus, the auditory information, i.e. the spoken names in this study, seems to support object-location binding. It will be interesting to examine whether auditory information that is used to encode location information will have a similar effect only on object location or whether it will also modulate object-location binding. If identity and location do not operate on the same level of representation, one could expect that identity memory is enhanced by such a location manipulation as well.

This novel audiovisual approach supports the binding of object-location and object-identity. Object-location-binding is particularly relevant for cartography, as maps do not represent spatial or graphical content in a separate but rather in an integrated way. Maps communicate semantic information that is geo-referenced and, thus, has clear locational quality. The reported findings, especially the effects of audiovisual names on the positional accuracy of recalled objects, might help to counteract spatial distortions that occur in a cognitive map based on indirect experience gathered from maps.

The observed effects of audiovisual map elements for cartographic communication should encourage cartographers to question the ‘graphic dominance’ in traditional map-making. Multimodal, especially audiovisual map construction, could be further developed in modern map-making and the potential of audiovisual communication for effective map use should be explored more deeply. In addition to exploring the effectiveness of audiovisual cartography, the question of how auditory map elements are processed is at the heart of understanding spatial memory processes. The targeted use of ‘map acoustics’ may further improve the quality of cognitive representations of geographic space.

## Supporting information

S1 File Anonymized raw data of 32 participants. (SAV)

S2 File Anonymized raw data of 36 participants. (SAV)
